# Saved in translation? Diversity shared in French and Dutch medieval literature

**DOI:** 10.1017/ehs.2026.10036

**Published:** 2026-02-02

**Authors:** Mike Kestemont, Folgert Karsdorp, Jean-Baptiste Camps, Remco Sleiderink, Anne Chao

**Affiliations:** 1 University of Antwerp, Antwerp, Belgium; 2 KNAW Meertens Institute, Amsterdam, the Netherlands; 3 École nationale des chartes (PSL), Paris, France; 4 National Tsing Hua University, Hsinchu, Taiwan

**Keywords:** cultural heritage, translation, medieval literature, unseen species models

## Abstract

Empirical studies often have to work with incomplete samples, with scholars rarely accounting for under-registration: in cultural heritage e.g. the age-long loss of artefacts can yield an under-estimation of the original richness of assemblages. Recently, it has been argued that unseen species models from ecology can estimate the unobserved diversity in cultural collections. We report an extension on *shared* diversity, i.e. the number of types that are common to two assemblages. As a case study, we use stories in medieval French and Dutch (ca. 1150–1450), which were frequently shared. We apply an established estimator (Chao-shared) with a novel bootstrap procedure. The estimator suggests that the surviving data underestimate the original number of shared stories: for example, when its source is no longer extant, a translation can no longer be identified as such. Interestingly, there is less evidence for the total loss of shared stories: precisely because of the redundancy caused by inter-vernacular translation, shared stories were less likely to be lost in both languages simultaneously. These results go beyond previous studies in that they provide more insight into the composition of the unobserved share of cultural diversity (instead of its mere size).

## Introduction

Empirical work across many research domains is commonly based on samples that were drawn from a larger population. Diversity (or complexity) is an important characteristic of such samples. An important and intuitive diversity metric is the number of unique types in an assemblage of token counts, its richness, although alternative approaches to quantifying diversity exist (Chao et al., 2014, Daly et al., 2018). In spite of significant registration effort and sensible sampling design, samples are typically incomplete: many types available in the original population might be missing (Chao, 1984). The observable diversity in a sample therefore commonly underestimates the true diversity of the underlying population. Consequently, correcting or de-biasing the diversity attested in samples has important applications across many disciplines, including ecology (Magurran, 1988), criminology (van der Heijden et al., 2014), and archaeology (Kaufman, 1998). In ecology, for instance, the issue of ‘unseen species’ has long been acknowledged (Fisher et al., 1943), because empirical field surveys of biodiversity tend to underestimate the true number of unique species inhabiting a given geographical area. (Animal species might be difficult to observe for a variety of reasons, for example, because they are shy, rare, or well camouflaged (Chao & Chiu, 2016)). A family of biostatistical methods known as ‘unseen species models’ has been developed to deal with this situation.

Recently, it has been argued that unseen species models find a relevant application in the study of cultural heritage (Kestemont et al., 2022). Work in this area is, by necessity, based on the historical record consisting of the material artefacts (such as books, paintings, or statues) that still survive today. However, such cultural assemblages have commonly sustained substantial material losses (Esch, 1985), resulting from deliberate destruction (e.g. book burning), unintended catastrophes (e.g. library fires), or more casual forms of gradual forgetting (e.g. resulting from diachronic changes in aesthetic appreciation). (A well-informed recent survey, specifically for the British Isles, has been published by (Milne, 2025)). Because of such losses, present-day heritage collections tend to underestimate the original diversity of the past human cultures that they represent. Recent studies have shown that unseen species models can provide a valid quantitative estimate of the cultural diversity that has not been attested in the available samples. Recent applications of this approach include lost historic literature (Martynenko, 2023), unregistered sailors from the East-Indian company (Wevers et al., 2022), or policing bias in historical arrest data (Karsdorp et al., 2024). Earlier studies in this area happened in numismatics (Esty, 1986).

In this paper, we turn to the problem of estimating the cultural richness (diversity) that is *shared* by two communities. Connected communities often overlap in their cultural expression because of processes of migration or social learning (e.g. translation) that result in cultural exchange. However, in the face of severe material losses, as is typical with historical records, the reliable estimation of the original, true size of this overlap or shared richness is prohibitively difficult. Here, we focus on the surviving corpus of medieval chivalric literature in Dutch and French (ca. 1150–1450). The texts documented in the surviving assemblages show a considerable overlap, mainly because many Middle Dutch texts were derived from Old and Middle French counterparts (which were more prestigious at the time). The fact that so many of these stories, in either language, did not survive makes it particularly challenging to estimate how many they originally shared, thus clouding our view of the cultural transfer between both communities. Here, we borrow an established method from ecology and use it to estimate the number of stories that these literatures originally shared.

The structure of this paper is as follows. We first introduce at greater length the cultural phenomenon of chivalric literature in North Western Europe during the medieval period, with special attention to the material text carriers on which this literature circulated and the later loss of these artefacts. Next, we introduce the case study and associated data used for this study, i.e. counts of the (translated) chivalric texts that survive in medieval French (*langue d’oïl*) and Dutch (*Middelnederlands*). Subsequently, we introduce the Chao1 estimator and explain how it can be applied to estimate the number of lost stories in a single assemblage; we go on to discuss Chao-shared, an extension of this method to estimate the unobserved diversity shared between two assemblages. A novel contribution is the bootstrap approach proposed to obtain confidence intervals for all estimands. Finally, we present the outcome of our analyses and confront the results with prior scholarly debates, in particular on the authenticity of Middle Dutch literature.

## Materials

### Medieval chivalric narratives

Chivalric and heroic stories, such as the courtly romances about King Arthur or the epics about Charlemagne, were an important component of the cultural production in Europe during the Middle Ages (Krueger, 2000). The twelfth century witnessed the emergence of secular written literature in the continent’s vernaculars. Frequently, chivalric stories were transmitted across linguistic communities via translations or adaptations. The stories about King Arthur are a case in point: originating in Celtic folklore, these were adapted into Old French by leading authors such as Chrétien de Troyes. His texts were subsequently adapted into Middle High German, such as Wolfram von Eschenbach’s *Parzival* (van Oostrom, 2006). As *lingua franca*, the prestigious French culture was a dominant force in these developments – the *romanz* language even literally lent its name to the newly emerged text variety as a whole (cf. ‘romance’). Many vernacular texts across the European continent thus derived from French source materials: in the case of Middle Dutch (*Middelnederlands*), for instance, a meaningful share of the extant texts were in fact based on French originals.

Before the introduction of the printing press in Europe, texts were manually copied by scribes onto handwritten text carriers, such as parchment or paper codices and scrolls (Kwakkel, 2018). The number of distinct witnesses that today still survive of a medieval text is often informally treated as a proxy for the historic market demand that existed for these texts. Such count data is nevertheless dangerous to take at face value because of the significant losses which these artefacts sustained. Based on surviving inventories of medieval libraries, book historians have estimated a survival rate of only ca. 9% for the general population of medieval manuscripts (Wijsman, 2010). In prior work, it has been argued that unseen species models from ecology offer a principled, quantitative framework to estimate how much of this literature has been lost (Kestemont et al., 2022). Based on the number of rare, poorly attested texts (that only survive in a few witnesses), biostatistical models can be used to estimate a lower bound on the number of texts that did not survive at all. We will discuss these methods in more detail below.

Here, we extend this research to a novel aspect of lost medieval literature: the stories that were shared across two medieval literatures. We specifically focus on stories that concurrently existed in the French and Dutch literary culture of the period. When it comes to stories that were shared between French and Dutch, for instance, the following estimands should be considered:

1. Surviving Dutch translations based on French originals, which are no longer extant;
2. Lost Dutch translations based on French originals, which are extant;
3. Lost Dutch translations based on French originals, which are no longer extant.

Taken together, these estimands can shed new light on the number of stories that must have been shared by two cultures, even if this is difficult to observe in the empirical data because of the historic loss of sources.

### Terminology

Because we tackle complex concepts from past cultural production, it is important to resort to a maximally precise terminology. In a recent collaboration aimed at establishing an international database of medieval textual traditions, an ontology was negotiated that we try to consistently adopt throughout this paper. By a story, we shall indicate an identifiable, unique sequence of events, fictitious or not (e.g. Perceval’s quest for the Holy Grail), conceived as independent of their manifestation in texts (Culler, 1981). Such a story can find a concrete expression in a text, which is by definition created in a specific language, through the authorship of a specific individual (e.g. Wolfram von Eschenbach’s Middle High German *Parzival*). Finally, we distinguish the witness, or a copy which records a concrete, material manifestation of the text (e.g. the fifteenth-century illuminated copy by Diebold Lauer’s workshop, nowadays kept under the shelfmark ‘Heidelberg, Universitätsbibliothek, Cod. Pal. germ. 339’). If a medieval witness nowadays survives as disjoint objects (or documents), such as book fragments scattered across different locales, we still count this set of documents as a single witness.

A dedicated publication (Christensen and Camps, 2025) discusses in more detail how these terms map onto other scholarly traditions and encoding standards, such as the Library Reference Model Object-Oriented (
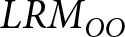
). Whereas the story and text are intangible abstractions, witnesses (and documents) refer to material heritage objects. Note that, in our terminology, stories can be shared across multiple languages, but texts never. Important for the present contribution is that the same story might have been realised in multiple texts (e.g. in different languages) that might be related or not: in the case of a translation, the target text explicitly derives from a pre-existing source text (in another language), but both would still be considered as realisations of the same underlying story. In our modelling approach, we treat stories as unique species, and we consider the two languages in which these stories have been realised as texts as distinct assemblages. The number of witnesses, in either language, refers to the number of extant copies that we can still observe for these texts in either assemblage, analogous to the notion of species sightings in ecological terms. Shared species are therefore stories that have (observed or unobserved) realisations as texts in both languages, whether they were translated from one another or not.

### Shared stories

The term ‘Middle Dutch’ refers to the amalgam of West-Germanic, vernacular dialects that were spoken in the Low Countries during the medieval period, ca. 1150–1450 (Van de Velde, 2024). Written literature is extant in this language from the middle of the twelfth century onwards (van Oostrom, 2006). Typically, the Middle Dutch period is said to run until ca. 1450 (until the introduction of the movable type printing press in the Low Countries) according to conventional periodisations. An important component of the Middle Dutch literature was the *ridderepiek* (Goossens, 2005, Caers & Kestemont, 2011, Caers, 2011), a corpus of chivalric stories that still has a great deal of prestige in neerlandophone culture. Where the earliest production of this text variety was concentrated in the Low Franconian region in the east (counties like Looz and Guelders), it shifted towards the West (the county of Flanders and the duchy of Brabant) as the thirteenth century progressed (Caers & Kestemont, 2011, Caers, 2011). *Ridderepiek* is more sparsely documented in the northern regions of the Low Countries, such as the County of Holland. To the present day, ca. 75 texts have survived in this text variety, surviving in ca. 150 fragmentary or (near-)complete witnesses. Famous examples include the *Roman van Walewein*, an originally Middle Dutch Arthurian romance, the *Roelantslied*, an adaptation of the French *Chanson de Roland*, and the *Historie van Troyen*, which Jacob van Maerlant translated from the *Roman de Troie*. A foundational inventory of this corpus can be found in a repertory by Kienhorst (Kienhorst, 1988), although a number of important, new discoveries have been made since then.

Because of processes of inter-vernacular exchange, literary heritage was to a meaningful extent shared in this period. A narrow majority of the surviving Middle Dutch texts (38/72) are known to derive in some way from French sources: the French *langue d’oïl* enjoyed an enormous cultural prestige in this period, and many originally French texts were translated into other vernaculars, such as Middle Dutch (Morato and Schoenaers, 2019). In some cases, Dutch texts even claim to be translations from French, probably to add to their cultural prestige, even if these assertions are sometimes demonstrably false (Besamusca, 2008, Sleiderink, 2010a). Although a majority of the surviving Middle Dutch texts are somehow associated with French sources, the nature of this relationship can range from very faithful translations to more creative adaptations (Gerritsen, 1988). Determining this can be challenging: what appears to be a creative adaptation of a known French text may in fact be a faithful translation of another French text that is now lost. In other cases, stories can be shared across these two literatures, even if no direct dependence has been attested. A complex case is the Middle Dutch *Karel ende Elegast* (Janssens, 2023): a very similar text must have existed in French, called the *Chanson de Basin. The *Basin*, however, has not survived and we only know it from other indirect evidence referencing it: this makes it difficult to assess whether the *Elegast* was directly based on this text. Because the protagonist carries a different name (*Basin* vs. *Elegast*), this is an example of a story that was shared, although the precise nature or the direction of the cultural exchange cannot be assessed.

For our analysis, we have reused previously collected abundance data for the chivalric and heroic texts that survive in French and Dutch (Kestemont et al., 2022): this data holds the number of handwritten witnesses per text that still survive today, which we treat as observation events, or ‘sightings’ in an ecological sense. Next, we aligned the datasets and paired the texts that shared the same underlying story, typically (but not exclusively) because the Dutch text was based on a French original. Thus, we obtain text attestation frequencies for each story in both languages, whether the text is known to have been translated or not.^1^ Some French originals were adapted into Dutch multiple times, such as the *Lancelot en prose* of which three independent adaptations in Middle Dutch survive (Gerritsen, 1988). In such cases, the number of sightings for the Middle Dutch adaptations, even if they constitute different texts in our terminology, was collapsed into a single cumulative count. Table 1 provides basic statistics on the two assemblages considered here.

**Table 1.** Diversity statistics for the chivalric subcorpora and the pooled result, including the single-assemblage Chao1 estimate (with 0.95 confidence intervals) of the total number of texts

	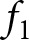	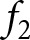	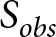	n	(lower)	Chao1	(upper)	Chao1(%)
Dutch	39.00	15.00	72.00	170.00	92.22	122.40	174.33	0.59
French	91.00	21.00	224.00	1579.00	351.09	421.04	520.96	0.53
Pooled	110.00	26.00	258.00	1749.00	411.34	490.56	596.46	0.53

Establishing these aligned counts comes with many challenges that should be made explicit. Discretisation, or determining whether two manuscripts offer a witness of the same text can be challenging – the same is true for assessing whether two texts share the same underlying story. Interestingly, similar issues arise in ecological surveys, where it can be equally difficult to establish whether two individuals belong to the same species or not (Daly et al., 2018). While many scribes produced relatively faithful copies of their exemplars, others intervened more actively in their source text, for instance, through the practice of compilation or abridgement. Such changes can be so profound that domain experts categorise the witness as a new text altogether. In general, we took the executive decision to stick with the existing categorisations of witnesses in the authoritative inventories, which we consulted. Many witnesses only survive in a fragmentary, heavily damaged form, adding to the complexity of identifying the recorded text, let alone whether the story also existed in the other vernacular (where many of the surviving witnesses are also incomplete). In the case of short fragments, we might also not be able to observe that two seemingly unrelated witnesses are in fact non-overlapping fragments of the same text. We must therefore account for the fact that this situation introduces some one-inflation in our data, or the relative over-counting of singletons (Böhning & Friedl, 2024).

**Figure 1.**
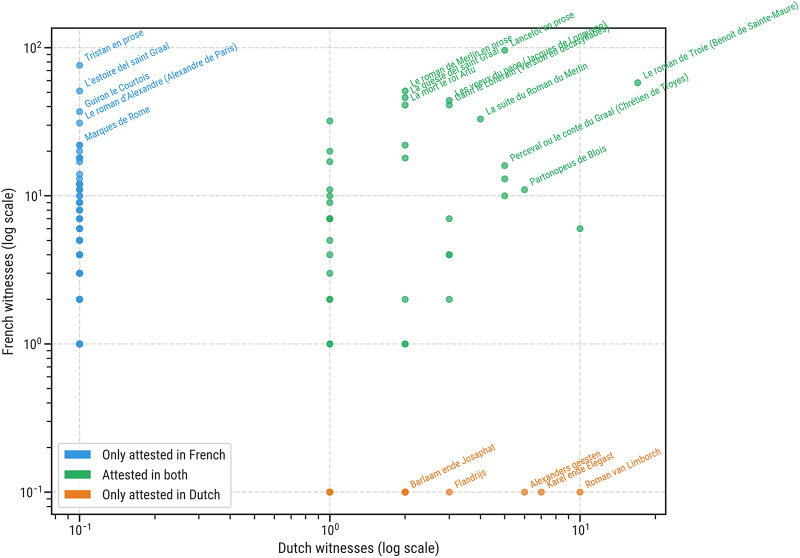
Text attestation patterns (i.e. the number of witnesses) for chivalric texts in Dutch and French traditions. Log-log scatter plot showing the number of witnesses per story in each language. Colours distinguish attestation patterns: Dutch-only (orange), French-only (blue), and both languages (green). Top-ranking titles in each category are annotated.

Intuitively, it would make sense to hypothesise a weak but positive correlation between the attestation counts of stories in either language. When we take the original, paired counts for all stories (i.e. including texts with a zero count in one of the assemblages), no positive correlation is evident (cf. Figure 1): well-attested stories in French literature are not necessarily well attested in Dutch (and vice versa). A non-parametric, directional Kendall’s 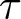 test revealed no significant positive correlation whatsoever (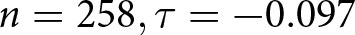, 
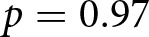
). On the contrary, some of the texts with the highest attestation counts in Dutch are in fact not translations (e.g. *Roman van Limborch*), with the notable exception of the evergreen *Roman de Troie* / *Historie van Troyen*, which appears to have been equally successful in either community. Likewise, a number of extremely popular texts in French, such as the *Tristan en prose*, are without an empirically traceable counterpart in Dutch. It remains unclear, of course, to which extent the post-medieval loss of witnesses is responsible for this. If we limit the test to the observably shared stories, i.e. stories that have been attested at least once in both languages, a mildly positive effect emerges: (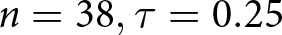, 
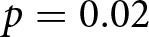
). This suggests that, while the empirical data does not suggest an obvious (positive) correlation, the pattern of source survival might be obstructing our understanding of this shared literary heritage.

## Methodology

First, we will briefly review the original Chao1 estimator for species richness in a single, ecological community, which can be extended to the problem of estimating the number of distinct species *shared* by two communities Chao-shared).^2^

### Chao1 estimator

Consider a single assemblage of types which are represented as a vector of counts (strictly positive integers). Let 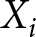 denote the abundance or frequency of the 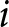
th type in the sample: 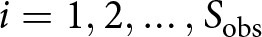, where 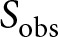 refers to the number of unique types observed in the sample and 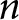 the cumulative number of tokens or sightings in the sample. In the case of undersampling, 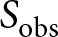 will underestimate the true number of distinct types in the underlying population (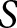, and the corresponding estimate 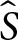). If we denote the number of unobserved species as 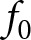 (and the corresponding estimate 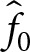), the original, true diversity of the population can be estimated as 
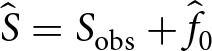
. Chao1 is an established estimator (Chao, 1984) to obtain a universally valid *lower bound* of 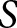, so that 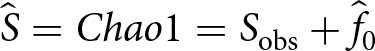, where 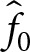 is defined in Eq. 1. The method adopts the perspective that a reliable lower bound is of more value than an inaccurate point estimate.

In prior work, we have more extensively argued the applicability of unseen species models, in particular Chao1, to abundance data that records the surviving witnesses of medieval texts.^3^ Chao1 is an estimator that can, in principle, be applied to any hyper-diverse assemblage that has been severely undersampled. The estimator makes minimal theoretical assumptions: we only assume that there exists an unknown number of types (stories or texts, in the present case) in an assemblage of entities (here, witnesses). We also assume that a sample of these entities has been independently drawn from an originally much larger collection and that each entity can be correctly classified to its type (i.e. each witness can be assigned to a story). Here, independent sampling means that the outcome of any sampled entity does not affect, nor does it reveal any information about the outcome of other sampled entities. Chao1 yields a universally valid lower bound for the true number of classes; the estimator is non-parametric and makes no assumptions about the species abundance distribution, meaning that the estimator also holds when the abundance or detection rates differ wildly across species types.

In this approach, rare types in the abundance vectors are of special relevance, as it was already argued by Alan Turing that sparsely documented types carry most information about the types which were not observed at all in a sample (Chao et al., 2017). Quantities of special interest below are 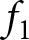 and 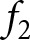, or the number of types in a sample that have an attestation frequency of 1 and 2, respectively – also called singletons and doubletons. For a sample with 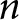 observations, Chao1 estimates the total number of species by adding the observed species richness 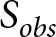 to an estimated number of unseen species, 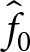, as follows:
(1)
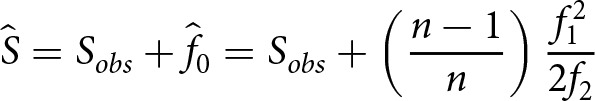


The method was originally derived as a lower bound from a moment inequality (Chao, 1984). When 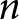 is large, removing the first term will have a negligible effect. Using a bootstrap approach, confidence intervals can be reported for these estimates to account for the considerable uncertainty in the procedure. The observation rate, or the survival ratio in the case of medieval texts, can be calculated as the following proportion: 
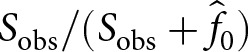
. Note that this ratio becomes an *upper bound* (as Chao1 provides a lower bound), i.e. this ratio indicates the *maximum* proportion of narratives that have survived (Kestemont et al., 2022).

**Figure 2.**
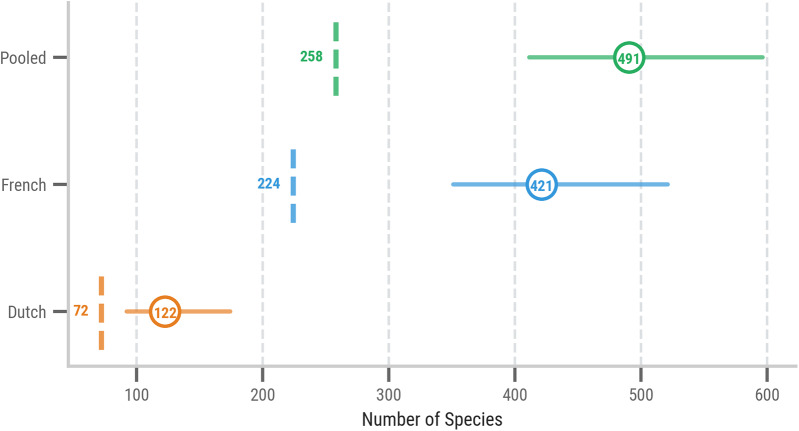
Single-assemblage Chao1 estimates: Dutch, French, and ‘pooled’, meaning the frequencies of each row are combined, including 0.95 confidence intervals and survival ratio for the central point estimate. Dashed horizontal lines (left) indicate observed diversity; encircled numbers reflect the debiased estimate (with CIs indicated via the horizontal line.).

In Table 1, we report the Chao1 estimates for the French and Dutch chivalric texts in isolation, and for the pooled data, which are visualized in Figure 2. (Note that these estimates deviate slightly from our earlier work, because the discretisation of texts follows a different logic here, for example, when the counts of parallel Dutch translations are collapsed.) In general, there is a meaningful difference in sample size across the two languages, but Chao1 suggests a very similar loss rate for both languages: in both French and Dutch, it is estimated that a maximum of roughly half of the texts are still known today. Prior research discussed the implications of this finding in the light of previous work in philology (van Oostrom, 2006, Kestemont and Karsdorp, 2019, 2020), which has often been more optimistic about the survival rate of medieval literature.

### Chao-shared estimator

From the aligned data, it becomes clear that some texts belong to stories which were shared across the two cultures. However, due to the heavy losses of witnesses on both sides, the present data in all likelihood severely underestimates the size of this shared repertoire (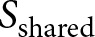). This term can be decomposed into:
(2)
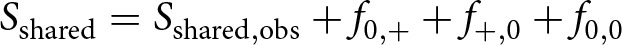


The first term (
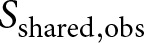
) indicates the number of stories that can today still be empirically observed to have been shared, i.e. the number of stories with a non-zero attestation count in both languages. This number however should be adjusted upwards by the estimands 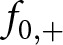, 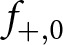 and 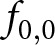, which each capture their own aspect of the – now lost, or ‘latent’ – shared diversity. Assume that the French assemblage is ordered first in the collection pair, so that 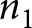 refers to the total number of empirical witnesses (tokens) in the French corpus and 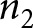 represents the cumulative number of Dutch witnesses. 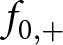 refers to the number of shared stories which are nowadays attested in Middle Dutch witnesses, but no longer in French:
(3)
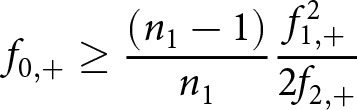


This quantity is of great interest, because many Middle Dutch texts are assumed to be translations that can no longer easily be identified as such, because the source text no longer survives in any witnesses.

Then, there is: 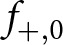:
(4)
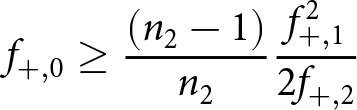


This is the number of French texts today, which are still attested in French witnesses, which were shared with Middle Dutch, even if no Middle Dutch witnesses are still extant.

Finally, there is one, even more abstract estimand, 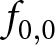:
(5)
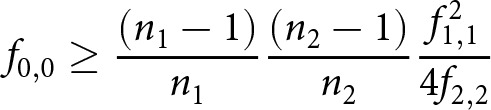


This quantity indicates the number of stories, which were originally shared, even if they can no longer be observed in either assemblage.

Readers will note the similarities of the aforementioned formulas to the conventional Chao1 estimator (Eq. 1): to estimate 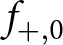, for example, we rely on the stories which were indeed shared with Middle Dutch, but which barely survived, i.e. as singletons (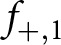) and doubletons (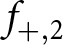). This information on the abundance of the most scarce and obscure texts in this category is then exploited to provide a lower bound estimate on the number of texts in this category which did not survive at all. Because all these equations ultimately provide lower-bound estimates, the same will be true of the combined estimate, which we consequently should write down as:
(6)
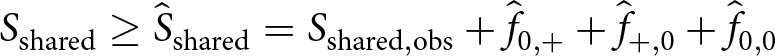


In the appendix, we present a novel bootstrap procedure which can be used to obtain confidence intervals on each of the estimands. Taking these into account is crucial, especially when working with smaller, heavily undersampled assemblages like ours, where there is considerable uncertainty in the estimates. Below, we will report upper and lower .95 percentiles for the obtained bootstrap values, yielding intervals that are not necessarily symmetric.

## Results

**Figure 3.**
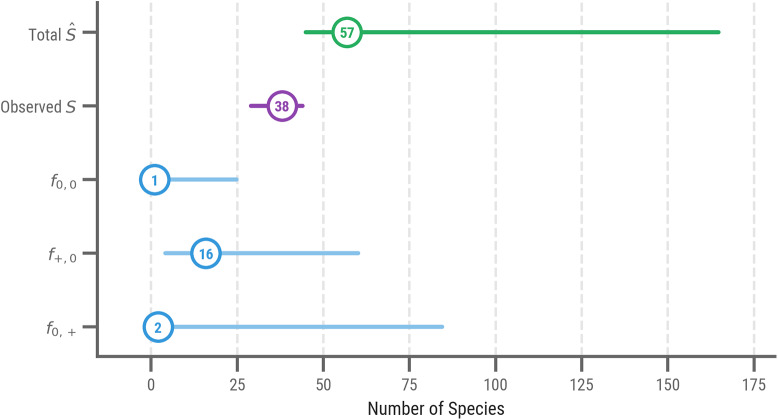
Visualisation of the (component) Chao-shared estimates for the chivalric corpus (for 1,000 bootstrap iterations, with circled point estimates and CIs as horizontal lines). Cf. Table 2.

In Table 2, we present the results of the shared richness estimator, including the 0.95 confidence intervals obtained through the bootstrap procedure. These estimates are also visualised more intuitively using a forest plot-like graph in Fig. 3. Overall, the results confirm the view that the number of stories which were originally shared by these two literary cultures is indeed underestimated by the still surviving, empirically observable data points. When discussing the constituent point estimates, however, we should take into account that (1) these are lower bounds, and that we should consider that these estimates might be fairly conservative; (2) there exist fairly wide confidence intervals around these estimates, which is probably due to the overall data sparsity that characterises the assemblages, as well as the considerable losses which either assemblage sustained.

**Table 2.** Chao-shared estimates for the chivalric corpus (for 1,000 bootstrap iterations)

**Quantity**	**Estimate**	**SE**	**95% LCL**	**95% UCL**
total ( 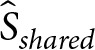 )	56.90	32.46	44.85	164.70
obs. shared	38.00	3.71	29.00	44.00
unobs. shared	18.90	27.24	8.01	169.43
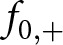	2.00	22.25	3.12	84.45
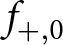	15.91	14.37	4.14	60.14
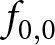	0.99	6.31	0.75	24.84

## Discussion

Our central estimate (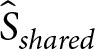) confirms that the number of chivalric stories which were originally shared by French and Dutch medieval literature is underestimated in the surviving sample (38 shared stories can now be observed): the point estimate suggests that originally at least ca. 57 stories were shared. The CI moreover shows that this might be a fairly conservative estimate in that there is more upside than downside potential for this quantity ([44.85–164.70]). This central estimate is valuable in its own respect because it sheds new light on the composition of the unobserved share for the population. For Middle Dutch literature, the standard Chao1 estimates that a minimum of about 50 stories were lost from the original population: Chao-shared estimates that a minimum of 17 of these lost stories (
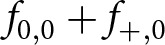
) – roughly a third (34%) – must have been shared. (The great majority of the texts relating those stories will have been translations.) This quantity is not negligible and should, well and truly, be accounted for in historical scholarship. Another way in which Chao-shared improves on the interpretability of this kind of research is that it can be naturally decomposed into a number of constituent estimands (
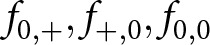
). These can help improve our understanding of why these shared stories have remained under-observed. Below, we will zoom in on each of these constituent estimands and attempt to connect it to existing scholarship in philology.

### Lost French pretexts (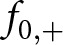)

The estimand 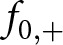 indicates that 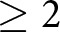 surviving stories in Middle Dutch had a French counterpart which is now lost altogether. The already mentioned *Karel ende Elegast* is probably one of these, because the French counterpart is no longer extant but has been attested otherwise. Another example where a lost French original has been attested via other ways is the *Roman de Torrez*, which survives in Middle Dutch translation but not in the French original – even though this original is known indirectly from a historic library inventory (Claassens, 2009). Interestingly, the estimator does not strongly suggest that there were many more cases like these, apart from the ones that we already knew about.

### Lost shared stories (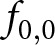)

We also have an estimate of shared stories that are now lost in both languages: 
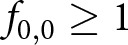
. It may seem very difficult to get an idea about the nature of these romances but there is evidence pointing in this area. There are, for example, early modern Dutch texts of which it is assumed that they are adaptations of lost medieval Dutch texts, and in some cases, these lost medieval Dutch texts may be based on a lost French original. Likewise, there exist German translations of now lost Middle Dutch texts, and again, some of these might find their origin in lost French texts. An intriguing case concerns *Johann aus dem Baumgarten* / *Joncker Jan wt den vergiere* (Scholz, 1991). The rhyming German text *Johann aus dem Baumgarten*, transmitted in a 15th c. manuscript, clearly mentions that it is derived from a rhyming Dutch, more specifically Flemish, source: ‘uz flemschen in unser dutsche sleht / zurbrochen rime machen reht’ (Duijvestijn, 1989, p. 153). The form and content of this now lost Dutch text can more or less be reconstructed on the basis of the German translation, but also a 16th c. Dutch prose adaptation, of which we have an edition dating to ca. 1590: *Joncker Jan wt den vergiere*. It must have been a 14th c. rhyming Dutch text about a foundling called ‘Jan’ (John) who is adopted by a German emperor, but who finds out in the end that his real parents are count Robert of Artois and a French princess. We may hypothesise that this now lost Dutch text was based on a French original (that was itself lost too). The plot of the reconstructed Dutch romance is similar to that of the French romance of *Richars li biau* – without this being the real source text – and it also has common features with the English romance *Sir Degaré*, which is in itself supposed to go back to a lost French romance or lai (Besamusca, 2003). In short, we hypothesise the existence of a French text *Jean du vergier which was translated into Dutch (and, on the basis of that, eventually also into German). Likely, the French text was intertextually related to other French texts like *Richars li biau* and the English *Sir Degaré* or its lost French source. Thus, texts that are only transmitted in one language may, in fact, have been translations of lost translations of lost texts. The example of the hypothetical *Jean du vergier and its ties with Dutch, German, and English literature, demonstrates that it would be worthwhile to consider more European languages in future scholarship. Latin, for instance, is an obvious omission in our work, even if chivalric literature was more rare in this language. An illustrative example is *Barlaam ende Josaphat*, a Middle Dutch text which is conventionally categorised as ‘ridderepiek’, although it borders on the text variety of hagiography, and might well have had a (currently unidentified) Latin*Vorlage* (Kienhorst, 1988).

### Lost Dutch translations (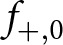)

The highest share of unobserved shared stories is represented by 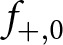, which is estimated to be 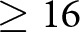, or the lower bound estimate on the number of lost Middle Dutch translations of French sources that are themselves still extant. External, additional evidence in this domain, however, is sparse and often indirect. Consider, for instance, the countal chamberlain John VI of Gistel: in 1417, the book collection of this Flemish nobleman included a ‘Dutch-language book speaking of the history of England and the Knight of the Lion’ (Caers, 2011). This seems to refer to a now lost Middle Dutch translation of the *Yvain* (or *Le chevalier au lion*) by Chrétien de Troyes. Since other Middle Dutch texts too reveal some familiarity with the Yvain matter, it is quite conceivable that poets were exposed to this narrative in a Middle Dutch rendition (Besamusca, 1994). In other cases, the evidence is less direct: an interesting archaeological find is the ‘Tristan slipper’, a fourteenth-century leather shoe cover that was found in Mechelen (Belgium) (Sleiderink, 2010b). This artefact features an unmistakable rendition of the famous orchard scene from Tristan and Ysolde. While this text must have circulated in the original French version in the Low Countries, no Middle Dutch translation survives from this period – disregarding the more eastern, Low Franconian *Tristant* (Goossens, 2005). The slipper, however, features a Dutch quotation that was likely extracted from a Middle Dutch translation that went missing.

A final category is the (somewhat notorious) Dutch intermediaries: previous scholars have argued that some medieval German texts have been translated from French but only via Dutch intermediaries, which are now lost. While there exist valid arguments for this hypothesis, it has remained controversial. Apart from the Arthurian texts discussed by Tilvis (van Oostrom, 2006), we should also mention *Schlacht von Alischanz*, a Charlemagne text that was translated from an existing French epic, *Chanson d’Aliscans* (Kienhorst, 1998, Bastert, 2010). The *Schlacht* survived in fragments that display a curious mix of High German and Low German dialectal features. However, scholars have also noted relics of a Middle Dutch lexical stratum in the text, with words such as *har unde tar* and *broecgurtel* (Leitzmann, 1920). These relicts suggest that the *Aliscans* might have impacted the German cultural area via a Middle Dutch intermediary.

### Implications

Understanding the transmission of cultural traits across individuals and communities is a central task in studying cultural change. Here, we focused on this task from the perspective of literary history and more specifically the inter-vernacular sharing (e.g. translation) of stories across two medieval literary systems (medieval French and Dutch). Modelling this process is complicated by the problematic survival of material sources, which can mask important interdependencies between these cultures, leading to an under-estimation of the original narrative richness of these literatures, but also their shared diversity. Above, we proposed the application of an established method (Chao-shared) from ecology to abundance counts that capture the (observed) abundance of texts in both languages. We discussed how the central estimate can be decomposed into three constituent estimates, which capture the intuition that shared stories cannot always be reliably observed, for instance, when either the source or translation has not survived in either language.

The resulting estimates broadly support the hypothesis that the observable, surviving data underestimate the shared diversity on several fronts. While this has been acknowledged by several studies in the past, not all scholars were equally convinced of the extent of this phenomenon. It is particularly meaningful to confront our results with the state of the art in Middle Dutch scholarship, where the authenticity or originality (*oorspronkelijkheid*) of this literature has been hotly debated. By ‘authentic’ or ‘original’, scholars typically mean that a text was originally written in Middle Dutch and has not been translated or adapted from another language, like French or Latin. In an ecological sense, the number of authentic texts could therefore be termed the ‘endogenic’ cultural diversity. As mentioned, a narrow majority of Middle Dutch texts should be considered translations (including more creative, less verbatim adaptations, which we do not distinguish as such in this context). For many Middle Dutch texts, however, it has so far not been possible to identify a French source. Earlier scholars, such as Gerritsen, were convinced that many of these texts derive from lost French exemplars (Gerritsen, 1988, p. 41). Besamusca has argued that it would be preconceived (*idée fixe*) to assume that all such Middle Dutch texts must derive from now lost French sources: he proposed the opposite view, i.e. to consider such texts, in the lack of additional evidence, as authentic or endogenic: ‘Wenn kein französisches Original nachzuweisen ist, gab es auch keines’ (Besamusca, 2008, p. 35).

The bootstrap approach presented above also yields, perhaps surprisingly, a confidence interval for the attested number of shared stories, even if this quantity is directly observable. (Due to the nature of the bootstrap, note that the CI will not necessarily include the observed value, even if this happens to be the case above.) From a statistical perspective, the CI reflects the uncertainty associated with the observed value: if similar sampling or data collection were repeated, the number of observed shared species would vary, and the CI provides the likely range of this variation. The resulting range ([29–44]) seems informative in the context of the 38 shared stories which we identified: it gives a sense of the variation one might expect if a different team of scholars were to repeat this exercise – i.e. not so much taking inventory of the available texts, but the alignment exercise involved in mapping the texts to stories. In some cases, for example, there is clear but indirect evidence for a French pretext. The Middle Dutch *Florigout*, for instance, features excessively many French proper nouns (like ‘Gardepont’) which point towards an earlier French stratum. This view sheds a new light on some likely translation glitches. In the following passage, a monkey is sitting in a tree and blows a horn when a company passes under his tree. His master, the aforementioned Gardepont, reacts with lots of joy:

Lude riep de felle here

HAHAHAHA, surleboye

Mijn symminkel hevet proye

Nu vernomen goet ende vet. (Heeroma, 1962, l. 3770–3773, p. 29)

(‘Loudly the evil lord shouted: “Hahaha, my monkey, Surleboye, now has spotted a good and fat prey”’.)

Apparently, the Dutch author thought that ‘Surleboye’ was the name of the monkey (Kuiper & Hendriks, 1993–2025). However, it seems more likely that *sur le bois* (‘in the tree’) in the French source text was the place from where the monkey had spotted his ‘prey’.

For other texts, the debate as to the authenticity has a stronger ideological dimension. Early scholars, such as Jonckbloet and Te Winkel, considered the *Roman van Moriaen* as a notable example of an ‘authentic’ or ‘original’ Middle Dutch Arthurian text (Wells, 1971). It is the very first text in Dutch literary history that features a non-white protagonist. In the wake of ideological criticism, the international attention for this text has surged, making it perhaps one of the most widely read Middle Dutch texts internationally at this moment (Heng, 2018). A source for the *Moriaen* has never been retrieved in French philology, adding to the (partly chauvinistic) claims that *Moriaen* is indeed an endogenic text (van Buuren and Gysseling, 1971). However, this Dutch-centric view has not gone uncontested in the international literature (Sparnaay, 1959). In the 19th century Gaston Paris already wrote: ‘Le fait qu’on n’a pas retrouvé l’original français ne prouve naturellement rien; nous avons vu bien des exemples de pertes semblables’ (Paris, 1888, p. 254). Nevertheless, if *Moriaen* was indeed a translation or adaptation of a now lost French source, it might be easier to explain that in Wolfram von Eschenbach’s *Parzival* the hero also has a son with a black mother, Feirefiz, a knight who was not completely black, but striped, black and white. Several scholars, including international researchers (Heng, 2018), nevertheless continue to insist on the likely originality of this ‘endogenic’ romance (Smith & Zemel, 2021).

## Figures and Tables

**Figure A1.**
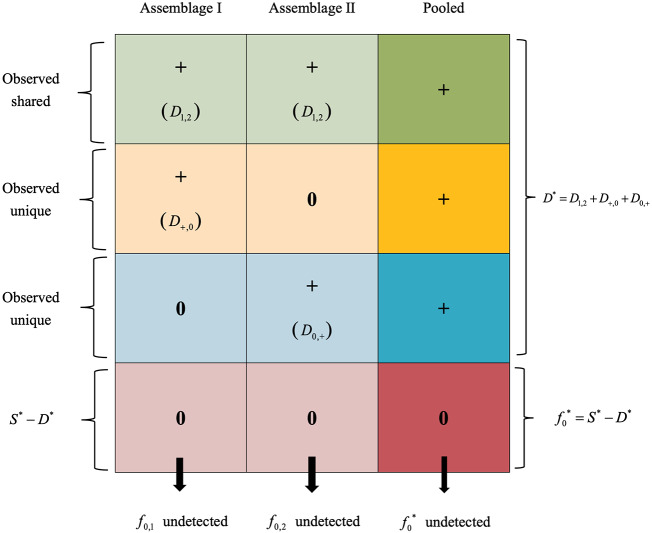
Bootstrap framework for shared species estimation showing the partition of species across two assemblages and their pooled combination. 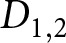, 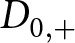, and 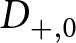 represent observed shared, unique to Assemblage II, and unique to Assemblage I species, respectively. 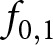, 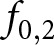, and 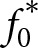 denote undetected species estimates for each assemblage and the pooled sample.

## Footnotes

1 Whereas a large portion of Dutch texts are known to be translations from French exemplars, no Dutch texts in this corpus are known to have been translated into French. Note, however, that the Chao-shared model is undirected and does not need to be informed about the direction of exchange; only the resulting shared richness is considered.

2 More accessible introductions of the methods have previously been published as online blogs (Karsdorp, 2022a, 2022b).

3 See, in particular, the supplementary information (SI) for Kestemont et al. (2022), available at https://www.science.org/doi/suppl/10.1126/science.abl7655/suppl_file/science.abl7655_sm.pdf.

## Research Transparency and Reproducibility

All software and data needed to replicate our analyses have been published online under a CC-BY licence: https://github.com/mikekestemont/shared-richness. The current and future releases of this repository are sustainable archived on Zenodo (https://doi.org/10.5281/zenodo.17236111). The central shared species estimator (with unit testing) is available from the *copia* package (v.0.1.6), commit number ‘4725e06’ (https://github.com/mikekestemont/copia).

## Appendix A. Shared species confidence intervals (bootstrap procedure)

We propose a bootstrap procedure for assessing the uncertainty of the estimates of , , , and total shared species between two assemblages. We resort to the bootstrap method proposed in the R package iNEXT.beta3D to assess the s.e. of the estimates of , , , and total shared species between two assemblages. Here, the total shared is the sum of observed, , , and . The associated 95% CI is also obtained. Assume a random sample I (species abundance vector with sample size ) was taken from Assemblage I with coverage estimate , undetected species estimate , and the Chao1 estimate . Next, assume a random sample II (species abundance vector with sample size ) was taken from Assemblage II with coverage estimate , undetected species estimate , and the Chao1 estimates . Finally, assume that in the data there were  observed shared species,  observed unique species in Sample II, and  observed unique species in Sample I. This is shown in Fig. A1.

The bootstrap procedures are briefly described in the following steps (see Fig. A1 for notation):

1. Consider the pooled sample with each species frequency being the sum of the two frequencies of the same species in the two samples. Then in the pooled sample there were  observed species. From the pooled sample, we first obtain the Chao1 species richness  and undetected species in the pooled assemblage . Then add  species to each assemblage as ‘undetected’ ones; see Fig. A1.
2. Then we construct two bootstrap assemblages: For bootstrap assemblage I: in addition to the observed data, we randomly select  species from zero cells (there are  of them) as those undetected species in Sample I. If , then replace  with  and select all  cells. Each selected species/cell is assigned to a relative abundance  in the bootstrap assemblage I.
3. For bootstrap assemblage II: in addition to the observed data, we randomly select  species from zero cells (there are  of them) as those undetected species in Sample II. If , then replace  with  and select all  cells. Each selected species/cell is assigned to a relative abundance  in the bootstrap assemblage II.
4. Within each sample, for any detected species, its relative abundance is coverage-based-adjusted according to the method in (Chao et al., 2015) so that the sum of the relative abundance of the detected species is equal to the coverage estimate in each sample.
5. After the two bootstrap assemblages are constructed, take a sample of size  from Bootstrap Assemblage I, and take a sample of size  from Bootstrap Assemblage II. Then compute bootstrap statistics (observed, ,  and ) based on the bootstrap sample.
6. Repeat Procedure (4)  times (, by default), then the sample s.e., based on the replicated values, is used as the approximate s.e. of each estimate for the current data. The corresponding 95% CI is computed using the percentile method: the lower and upper confidence limits are the 2.5th and 97.5th percentiles of the  bootstrap estimates, respectively.

Each 95% CI is computed using bootstrap percentiles rather than symmetric intervals. This percentile-based approach naturally handles the constraint that estimates should be non-negative and provides more appropriate confidence intervals for skewed distributions commonly encountered in (shared) species estimation. An advantage of this bootstrap method is that it can be directly generalised to multiple assemblages and does not rely on normality assumptions. The number of bootstrap replications should be at least 200, preferably 1000, for more accurate results.

## Appendix B. Shared stories in French and Dutch medieval texts

Below are aligned attestation counts for (shared) stories across Dutch and French medieval chivalric texts. If multiple independent translations or adaptations exist, the components have been joined with a semi-colon (;). Composite texts are indicated with a hyphen (-). These counts are largely based on the *Forgotten books* data (Kestemont et al., 2022), but with some minor emendations.

**Table:**

French text	Dutch text(s)	French	Dutch
Aimeri de Narbonne		6	0
Aiol	Limburgse Aiol (1); Vlaamse Aiol (2)	1	2
Aiquin		1	0
	Alexandercompilatie [Antwerp, UL, MAG-P 64.19]	0	1
	Alexanders geesten	0	6
Aliscans		18	0
Amadas et Ydoine		2	0
Ami et Amile (Version continentale en décasyllabes)		1	0
Ami et Amile (Version de Lille)		1	0
Ami et Amile (Version en alexandrins)		5	0
Ami et Amile (Version en prose du XIIIe s.)		1	0
Anseïs de Carthage		7	0
Anseïs de Gascogne		5	0
Apollonius de Tyr (version du XIIe siècle)		1	0
Apollonius de Tyr (version en prose I)		1	0
Apollonius de Tyr (version en prose II)		5	0
Apollonius de Tyr (version en prose III)		1	0
Apollonius de Tyr (version en prose III)		1	0
Apollonius de Tyr (version en prose V)		1	0
Artus de Bretagne		10	0
Aspremont	Roman van Aspremont [= Ongeïdentificeerd 5]	20	1
Athis et Prophilias (Alexandre de Paris)		12	0
Auberi le Bourguignon	Aubri de Borgengoen	7	1
Aye d’Avignon		4	0
	Barlaam ende Josaphat	0	2
Baudouin de Sebourc	Boudewijn van Seborch	2	2
Beaudous (Robert de Blois)		1	0
Berinus (Versions en prose)		5	0
Berte aus grans piés (Adenet le Roi)	Beerte metten breeden voeten	9	1
Beuve d’Aigremont		3	0
Beuve de Hantone (Version continentale I)		3	0
Beuve de Hantone (Version continentale II)		3	0
Beuve de Hantone (Version continentale III)	Boeve van Hamtone	3	1
Beuve de Hantone (Version en prose)		1	0
Blancandin ou l’Orgueilleuse d’amour (Version en vers)		5	0
Blancandin ou l’Orgueilleuse d’amour (Version longue en prose)		1	0
Brun de la Montagne		1	0
Buevon de Conmarchis (Adenet le Roi)		1	0
Chanson de Roland	Roelantslied	10	5
Chevalier au cygne (remaniement)		2	0
Chevalier au cygne, en prose		1	0
Ciperis de Vignevaux		1	0
Clarisse et Florent (en décasyllabes)		1	0
Cleomadés (Adenet le Roi)		14	0
Cleriadus et Meliadice		9	0
Cligés (Chrétien de Troyes)		11	0
Conquête de Jérusalem		12	0
Continuation de Jérusalem		1	0
Cristal et Clarie		1	0
Deuxième continuation du Conte du Graal (Wauchier de Denain)		11	0
Doon de la Roche		2	0
Doon de Mayence		4	0
Dou chevalier a l’espee		1	0
Du chevalier as deus espees		1	0
Durmart le Galois		2	0
Eledus et Serene		1	0
Elie de Saint Gilles		1	0
Enfances Godefroi	Godevaerts kintshede - Roman van Antiochië	10	1
Eracle (Gautier d’Arras)		4	0
Erec et Enide (Chrétien de Troyes)		11	0
Escanor (Girart d’Amiens)		1	0
Esclarmonde (en alexandrins)		1	0
Esclarmonde (en décasyllabes)		1	0
Fergus	Roman van Ferguut	2	1
Fierabras		11	0
	Fierabras	0	1
	Flandrijs	0	3
Floire et Blancheflor (Version I dite aristocratique)	Floris ende Blancefloer; Floyris ende Blantseflur	4	3
Floire et Blancheflor (Version II dite populaire)		1	0
Floovant	Floovent	2	1
	Florent ende Durant	0	1
Florent et Octavien (Version en alexandrins)		1	0
Florent et Octavien (Version en octosyllabes)		1	0
Floriant et Florete (Version en vers)		1	0
	Florigout	0	1
Florimont (Aimon de Varennes)	Roman van Florimont	17	1
Floris et Lyriopé (Robert de Blois)		2	0
Folque de Candie (Herbert le Duc de Dammartin)		17	0
Galeran de Bretagne (Renaut)		1	0
Garin de Monglane (Version en vers)	Garijn van Montglavie	1	1
Garin le Loherain (Version en décasyllabes)	Roman der Lorreinen (I-II)	41	3
Garin le Loherain (Version en prose)		1	0
Gaufrey		1	0
Gaydon		3	0
Girart de Roussillon (Jean Wauquelin)		2	0
Girart de Roussillon (Version en alexandrins)		3	0
Girart de Roussillon (Version en décasyllabes)		5	0
Girart de Vienne (Bertrand de Bar-sur-Aube)	Geraert van Viane	7	1
Girbert de Metz	Roman der Lorreinen [H58]	32	1
Gormont et Isembart	Loyhier ende Malaert	1	2
Gui de Bourgogne		3	0
Gui de Nanteuil		4	0
Gui de Warwick (Version en prose)		2	0
Gui de Warwick (Version en vers)		13	0
Guibert d’Andrenas		4	0
Guillaume de Palerne (Version en vers)		1	0
Guiron le Courtois		37	0
	Gwidekijn van Sassen	0	1
Hernaut de Beaulande (Version en prose)		1	0
Hernaut de Beaulande (Version en vers)		1	0
Hervis de Metz		5	0
Hugues Capet		1	0
Hunbaut (Version originale)		1	0
Huon d’Auvergne		4	0
Huon de Bordeaux (Version en alexandrins)		1	0
Huon de Bordeaux (Version en décasyllabes)	Huge van Bordeus	4	1
Ille et Galeron (Gautier d’Arras)		2	0
Jehan et Blonde (Philippe de Remi père)		1	0
Joufroi de Poitiers		1	0
Jourdain de Blaye (en décasyllabes)	Jourdein van Blaves	1	1
Kanor		6	0
	Karel ende Elegast	0	7
L’atre perilleux		3	0
L’entree d’Espagne		3	0
L’Escoufle (Jean Renart)		2	0
L’estoire del saint Graal		51	0
L’istoire de la Chastelaine du vergier et de Tristan le chevalier		1	0
La bataille Loquifer		11	0
La belle Hélène de Constantinople (Version rimée)		3	0
La chanson d’Antioche (Version du XIIe siècle)	Godevaerts kintshede - Roman van Antiochië	11	1
La chanson de Croissant (Version II)		1	0
La chanson de Florence de Rome		3	0
La chanson de Godin		1	0
La chanson des Saisnes (Jean Bodel)		3	0
La chastelaine de Vergi	Borchgravinne van Vergi (1); Borchgravinne van Vergi (2)	22	2
La chevalerie Vivien		12	0
La demoiselle a la mule (Paien de Maisieres)		1	0
La destruction de Rome (Version I)		2	0
La folie Tristan de Berne		2	0
La mort Aymeri de Narbonne		4	0
La mort de Maugis		1	0
La mort le roi Artu	Arturs doet	41	2
La prise d’Orange		9	0
La prise de Cordres et de Sebille		1	0
La queste del saint Graal	Queeste van den grale	46	2
La suite du Roman du Merlin	Merlijn-continuatie	33	4
La vengeance Raguidel (Raoul de Houdenc)	Wrake van Ragisel	4	3
	Laidoen	0	1
	Lanceloet en het hert met de witte voet	0	1
Lancelot en prose	Lantsloot van der Haghedochte; Proza-Lancelot; Roman van Lancelot	96	5
Lancelot ou le Chevalier de la charrette (Chrétien de Troyes)		8	0
Le bastard de Bouillon		1	0
Le bel inconnu (Renaut de Beaujeu)		1	0
Le charroi de Nimes		8	0
Le chevalier du papegau		1	0
Le couronnement de Louis		9	0
Le donnei des amanz		1	0
Le fuerre de Gadres (Eustache)		4	0
Le livre d’Artus		1	0
Le livre de Flourimont et de Helaine		1	0
Le moniage Guillaume (Version longue en vers II)	Willem van Oringen	7	1
Le moniage Rainouart		9	0
Le roman d’Abladane		1	0
Le roman d’Alexandre (Alexandre de Paris)		31	0
Le roman d’Alexandre en prose		18	0
Le roman d’Auberon		1	0
Le roman d’Eneas		9	0
Le roman d’Erec en prose (Version interpolée)		1	0
Le roman de Balain		3	0
Le roman de Cardenois		1	0
Le roman de Guillaume d’Angleterre (Chretien)		2	0
Le roman de Joseph d’Arimathie (Robert de Boron)		1	0
Le roman de Joseph d’Arimathie en prose	Historie van den Grale - Merlijns boec	18	2
Le roman de la poire (Tibaut)		3	0
Le roman de Laurin		9	0
Le roman de Merlin (Robert de Boron)		1	0
Le roman de Merlin en prose	Historie van den Grale - Merlijns boec	51	2
Le roman de Silence (Heldris de Cornouailles)		1	0
Le roman de Thebes		6	0
Le roman de Troie (Benoit de Sainte-Maure)	Historie van Troyen (Maerlant); Trojeroman (Segher)	58	17
Le roman des fils du roi Constant (Baudouin Butor)		1	0
Le roman du Castelain de Couci et de la dame de Fayel	Borchgrave van Couchi	2	3
Le roman du roi Artus (Rustichello da Pisa)		20	0
Le romans du conte d’Anjou (Jean Maillart)		3	0
Le romanz du reis Yder		1	0
Le roumanz de Claris et Laris		1	0
Le siege de Barbastre		6	0
Les aventures des Bruns (Rustichello da Pisa)		1	0
Les chétifs		6	0
Les enfances Garin de Monglane		1	0
Les enfances Guillaume		7	0
Les enfances Ogier (Adenet le Roi)		7	0
Les enfances Renier		1	0
Les enfances Vivien		9	0
Les faicts et conquestes d’Alexandre le Grand (Wauquelin)		2	0
Les Narbonnais		5	0
Les voeux du paon (Jacques de Longuyon)	Roman van Cassamus	44	3
	[Eén ridder, twee vrouwen]	0	1
Lion de Bourges (Version en alexandrins)		1	0
Mainet		1	0
Manekine (Philippe de Remi père)		1	0
Marques de Rome		22	0
Maugis d’Aigremont (Version en vers)	Madelgijs	6	10
Meliacin (Girart d’Amiens)		6	0
Melior		1	0
Melusine (Coudrette)		6	0
Melusine ou la noble histoire de Lusignan (Jean d’Arras)		6	0
Melyador (Jean Froissart)		2	0
Meraugis de Portlesguez (Raoul de Houdenc)		5	0
Naissance du chevalier au cygne (Beatrix)		8	0
	Nevelingenlied	0	1
Ogier le Danois (Version en alexandrins)		4	0
Ogier le Danois (Version française en décasyllabes)	Ogier van Denemarken	7	3
	Heinric en Claredamie [= Ongeïdentificeerd 1]	0	1
	Koning Ghaleane [= Ongeïdentificeerd 2]	0	1
	Het verraad van Pepijn [= Ongeïdentificeerd 3]	0	1
	Ongeïdentificeerd 4	0	1
	Wybeert van Andernaken [= Ongeïdentificeerd 6]	0	1
	Roman van Cassant [= Ongeïdentificeerd 7]	0	1
	Godevaert metten baerde [= Ongeïdentificeerd 8]	0	1
Orson de Beauvais		1	0
Otinel		4	0
Paris et Vienne (Pierre de La Cépède)		5	0
Paris et Vienne (version abrégée)		1	0
Parise la Duchesse		1	0
Partonopeus de Blois	Parthonopeus van Bloys	11	6
Perceval de Didot		2	0
Perceval ou le conte du Graal (Chrétien de Troyes)	Roman van Perchevael	16	5
Perlesvaus		9	0
Ponthus et Sidoine		22	0
Quatrième continuation du Conte du Graal		18	0
Raoul de Cambrai		2	0
Reinbert		1	0
Renaut de Montauban	Renout van Montalbaen	13	5
Renaut de Montauban (version en prose)		7	0
Retour du Cornumarant		9	0
	Roman van den riddere metter mouwe	0	2
Richars li biaus		1	0
	Roman van Caesar	0	2
	Roman van Iechemas	0	1
	Roman van Limborch	0	10
	Roman van Moriaen	0	2
	Roman van Torec	0	1
	Roman van Walewein	0	2
	Roman van Saladin	0	1
	Seghelijn van Jherusalem	0	1
	Sibeli en Aetsaert	0	2
Sone de Nansay		1	0
Syracon		1	0
Theseus de Cologne (Version en alexandrins)		2	0
Theseus de Cologne (Version en prose)		1	0
Tristan (Beroul)		1	0
Tristan (Thomas)	Nederfrankische Tristant	5	1
Tristan de Nanteuil		1	0
Tristan en prose		76	0
Troisième continuation du Conte du Graal (Manessier)		10	0
	Valentijn ende Nameloos	0	2
	Van den bere Wisselau	0	1
Vivien de Monbranc		1	0
Waldef		1	0
	Walewein ende Keye	0	1
	Willem van Oringen II	0	1
Wistasse le Moine		1	0
Yde et Olive		1	0
Yon ou la Vengeance Fromondin		1	0
Yonnet de Metz (remaniement en vers)		1	0
Ysaïe le Triste		1	0
Yvain ou le Chevalier au lion (Chrétien de Troyes)		12	0

## References

[ref1] Bastert, B. (2010). *Helden als Heilige: ‘Chanson de geste’ – Rezeption im deutschsprachigen Raum*. Max Niemeyer Verlag.

[ref2] Besamusca, B. (1994). Die Rezeption von Chrétien’s “Yvain” in den Niederlanden. In Ertzdorff X. v. (Ed.), *Die Romane von dem Ritter mit dem Löwen* (Chloe: Beihefte zum Daphnis, Vol. 20 pp. 353–368). Rodopi.

[ref3] Besamusca, B. (2003). The Book of Lancelot: The Middle Dutch Lancelot compilation and the medieval tradition of narrative cycles (T. Summerfield, Trans.). Brewer.

[ref4] Besamusca, B. (2008). Unidentifizierte mittelniederländische karlsepen: die idée fixe der mittelniederländischen Literatur als Übersetzungsliteratur. In H.-J. Ziegeler (Ed.), *Encomia Deutsch* (Vol. 1). V & R unipress.

[ref5] Böhning, D., & Friedl, H. (2024). One-inflation and zero-truncation count data modelling revisited with a view on horvitz–thompson estimation of population size. *International Statistical Review* 92, 406–430. 10.1111/insr.12570

[ref6] Caers, B. (2011). Een *buchelin inn flemische*. Over ontstaan en verspreiding van de ridderepiek in de Nederlanden (ca. 1150–1450). *Tijdschrift voor Nederlandse Taal- en Letterkunde* 127(3), 233–251.

[ref7] Caers, B., & Kestemont, M. (2011). De datering van de Middelnederlandse ridderepiek. *Verslagen en Mededelingen van de Koninklijke Academie Voor Nederlandse Taal- en Letterkunde*, 121(1), 1–59.

[ref8] Chao, A. (1984). Nonparametric Estimation of the Number of Classes in a Population. *Scandinavian Journal of Statistics*, 11(4), 265–270.

[ref9] Chao, A., & Chiu, C.-H. (2016). Species richness: Estimation and comparison. In *Wiley StatsRef: Statistics Reference Online* (pp. 1–26). Wiley. 10.1002/9781118445112.stat03432.pub2

[ref10] Chao, A., Chiu, C.-H., Colwell, R. K., Magnago, L. F. S., Chazdon, R. L., & Gotelli, N. J. (2017). Deciphering the Enigma of Undetected Species, Phylogenetic, and Functional Diversity Based on Good–Turing Theory. *Ecology*, 98(11), 2914–2929. 28869780 10.1002/ecy.2000

[ref11] Chao, A., Gotelli, N. J., Hsieh, T. C., Sander, E. L., Ma, K. H., Colwell, R. K., & Ellison, A. M. (2014). Rarefaction and Extrapolation with Hill Numbers: A Framework for Sampling and Estimation in Species Diversity Studies. *Ecological Monographs*, 84(1), 45–67.

[ref12] Chao, A., Hsieh, T. C., Chazdon, R. L., Colwell, R. K., & Gotelli, N. J. (2015). Unveiling the species-rank abundance distribution by generalizing the Good–Turing sample coverage theory. *Ecology*, 96(5), 1189–1201. 26236834 10.1890/14-0550.1

[ref13] Christensen, K., & Camps, J.-B., (2025). Greening your database of literary works: How to avoid reinventing vocabularies, in favor of sustainable, reusable models. In *DH2025 Book of Abstracts* ADHO. Lisbon, Portugal: ADHO.

[ref14] Claassens, G. H. M. (2009). De Torrez à Torec, un roman en moyen néerlandais et sa source inconnue en ancien français. In Goyens, M., & Verbeke, W. (Eds.), *Lors est ce jour grant joie née: Essais de langue et de littérature françaises du Moyen Âge* pp. (159–175). Leuven University Press.

[ref15] Culler, J. (1981). Story and discourse in the analysis of narrative. *The Pursuit of Signs: Semiotics, Literature, Deconstruction*, 169, 169–187.

[ref16] Daly, A., Baetens, J., & De Baets, B. (2018). Ecological diversity: measuring the unmeasurable. *Mathematics*, 6(7), 119.

[ref17] Duijvestijn, B. W. T. (1989). Middelnederlandse literatuur in Duitse overlevering: Een arbeidsveld voor neerlandici. In van Oostrom, F. P., & Willaert, F. (Eds.), *De studie van de Middelnederlandse letterkunde: Stand en toekomst. Symposium Antwerpen 22–24 September 1988* (pp. 153–168). Verloren.

[ref18] Esch, A. (1985). Überlieferungschance und Überlieferungszufall als methodisches problem des historikers. *Historische Zeitschrift*, 240(3), 529–570.

[ref19] Esty, W. W. (1986). Estimation of the Size of a Coinage: A Survey and Comparison of Methods. *The Numismatic Chronicle*, 146, 185–215.

[ref20] Fisher, R., Corbet, A. S., & Williams, C. (1943). The relation between the number of species and the number of individuals in a random sample of an animal pulation. *The Journal of Animal Ecology*, 12(1), 42–58.

[ref21] Gerritsen, W. (Ed). (1988). Vertalingen van Oudfranse literaire werken in het Middelnederlands. In R. E. V. Stuip (Ed.), *Franse Literatuur van de middeleeuwen*. (pp. 184–207). Muiderberg: Coutinho.

[ref22] Goossens, J. (2005). De schamele resten van de vroege Rijn-Maaslandse epiek. *Verslagen en Mededelingen van de Koninklijke Academie Voor Nederlandse taal- en letterkunde* 115, 53–72.

[ref23] Heeroma, K. H. (editor). (1962). *Florigout. Fragmenten van een 14de-Eeuws ridderverhaal*. Brill.

[ref24] Heng, G. (2018). *The Invention of Race in The European Middle Ages*. Cambridge University Press.

[ref25] Janssens, J. (2023). *Vertellen is een kunst. De Geschiedenis van Karel Ende Elegast*. Sterck & De Vreese.

[ref26] Karsdorp, F. (2022a). *Demystifying Chao1 With Good-Turing*. Blog post.

[ref27] Karsdorp, F. (2022b). *Estimating Unseen Shared Cultural Diversity*. Blog post.

[ref28] Karsdorp, F., Kestemont, M., & De Koster M. (2024). Beyond the register: demographic modeling of arrest patterns in 1879-1880 Brussels. In *CHR 2024: Computational Humanities Research 2024*, (Vol. 3834, pp. 265–281). CEUR Workshop Proceedings, Aarhus.

[ref29] Kaufman, D. (1998). Measuring Archaeological Diversity: An Application of the Jackknife Technique. *American Antiquity*, 63(1), 73–85.

[ref30] Kestemont, M., & Karsdorp, F. (2019). Het Atlantis van de Middelnederlandse ridderepiek. Een schatting van het tekstverlies met methodes uit de ecodiversiteit. *Spiegel der Letteren*, 61(3), 271–290.

[ref31] Kestemont, M., & Karsdorp, F. (2020). Estimating the loss of medieval literature with an unseen species model from Ecodiversity. In *Proceedings of the Workshop on Computational Humanities Research (CHR 2020)*, (Vol. 2723, pp. 44–55.) CEUR Workshop Proceedings, Amsterdam, The Netherlands.

[ref32] Kestemont, M., Karsdorp, F., de Bruijn, E., Driscoll, M., Kapitan, K. A., Macháin, P. O., Sawyer, D., Sleiderink, R., & Chao, A. (2022). Forgotten Books: The Application of Unseen Species Models to the Survival of Culture. *Science*, 375(6582), 765–769. 35175807 10.1126/science.abl7655

[ref33] Kienhorst, H. (1988). *De handschriften van de Middelnederlandse ridderepiek. Een codicologische beschrijving*. Sub Rosa, 9 Deventer Studiën.

[ref34] Kienhorst, H. (1998). Fragment van een onbekende Middelnederlandse ridderroman over Willem van Oringen. *Tijdschrift Voor Nederlandse Taal- en Letterkunde*, 114, 125–135.

[ref35] Krueger, R. L. (Ed.). (2000). *The Cambridge Companion to Medieval Romance*. Cambridge Companions to Literature. Cambridge University Press.

[ref36] Kuiper, W., & Hendriks, H. (Eds.). (1993–2025). Repertorium van Eigennamen in Middelnederlandse Literaire Teksten [REMLT].

[ref37] Kwakkel, E. (2018). *Books Before Print*. Amsterdam University Press.

[ref38] Leitzmann, A. (1920). Die kitzinger bruchstücke der schlacht von Alischanz. *Zeitschrift für Deutsche Philologie*, 48, 96–114.

[ref39] Magurran, A. E. (1988). *Ecological Diversity and Its Measurement*. Princeton, N.J, Princeton University Press.

[ref40] Martynenko, A. (2023). Unread, Yet Preserved: A Case Study on Survival of the 19th-Century Printed Poetry. *Literatura: Teoría, Historia, Crítica*, 25(2), 192–214.

[ref41] Milne, K. A. (2025). *The Destruction of Medieval Manuscripts in England. Institutional Collections*. Oxford University Press.

[ref42] Morato, N., & Schoenaers, D. (Eds). (2019). *Medieval Francophone Literary Culture Outside France*. Brepols.

[ref43] Paris, G. (1888). Romans en vers du cycle de la table ronde. *Suite du quatorzième siècle*. (Vol. 30, pp. 1–270). Imprimerie Nationale: Paris.

[ref44] Scholz, M. G. (1991). Johann aus dem Baumgarten und Joncker Jan wt den vergiere: Eine Skizze. Im Anhang: Transkription des Joncker Jan nach dem Amsterdamer Druck. In Haug, W., & Wachinger, B. (Eds.), *Positionen des Romans im späten Mittelalter* (Fortuna Vitrea, No. 1, pp. 146–232). Max Niemeyer Verlag.

[ref45] Sleiderink, R. (2010a). From francophile to francophobe: The changing attitude of medieval Dutch authors towards French literature. In Kleinhenz, C., & Busby, K. (Eds.), *Medieval multilingualism: The Francophone world and its neighbours* (Medieval Texts and Cultures of Northern Europe, pp. 127–143). Brepols.

[ref46] Sleiderink, R. (2010b). Op zoek naar Middelnederlandse literatuur in het Mechelen van de veertiende eeuw. Een tussenbalans. *Handelingen van de Koninklijke Kring voor Oudheidkunde*. *Letteren en Kunst van Mechelen*, 114, 31–54.

[ref47] Smith, S., & Zemel, R. (2021). Indigenous Arthurian romances: Walewein, Moriaen, Ridder metter mouwen, Walewein ende Keye, Lanceloet en het hert met de witte voet. In Besamusca, B., & Brandsma, F. (Eds.), *The Arthur of the Low Countries: The Arthurian legend in Dutch and Flemish literature* (Arthurian Literature in the Middle Ages, pp. 113–146). University of Wales Press.

[ref48] Sparnaay, H. (1959). The Dutch romances. In Loomis, R. S. (Ed.), *Arthurian literature in the Middle Ages: A collaborative history* (pp. 443–462). Clarendon Press.

[ref49] van Buuren, H., & Gysseling, M. (Eds.). (1971). *Moriaen*. W. J. Thieme & Cie.

[ref50] Van de Velde, F. (2024). Dutch. In Kürschner, S., & Dammel, A. (Eds.). *The Oxford encyclopedia of Germanic linguistics* (pp. 1–24). Oxford University Press.

[ref51] van der Heijden, P. G. M., Cruyff, M., & Böhning, D. (2014). *Capture Recapture to Estimate Criminal Populations*. Springer, pp. 267–276.

[ref52] van Oostrom, F. (2006). *Stemmen op schrift. Geschiedenis van de Nederlandse Literatuur van het Begin tot 1300*. Prometheus.

[ref53] Wells, D. (1971). The Middle Dutch Moriaen, Wolfram von Eschenbach’s Parzival, and Medieval Tradition. *Studia Neerlandica* 2, 1–86.

[ref54] Wevers, M., Karsdorp, F, and van Lottum, J. (Eds.). (2022). What shall we do with the unseen sailor? estimating the size of the dutch east india company using an unseen species model. In (Eds.), *Proceedings of the Computational Humanities Research Conference 2022, CHR 2022*, Vol. 3290 CEUR-WS.org CEUR-WS.org *Antwerp, Belgium*, December 12-14.

[ref55] Wijsman, H. (2010). *Luxury Bound. Illustrated Manuscript Production and Noble and Princely Book Ownership in the Burgundian Netherlands.1400–1550*. Brepols.

